# Genome sequencing of human *in vitro* fertilisation embryos for pathogenic variation screening

**DOI:** 10.1038/s41598-020-60704-0

**Published:** 2020-03-02

**Authors:** Nicholas M. Murphy, Tanya S. Samarasekera, Lisa Macaskill, Jayne Mullen, Luk J. F. Rombauts

**Affiliations:** 1 Genetic Technologies Ltd., Victoria, Australia; 2 Monash IVF, Clayton, Victoria, Australia; 3 GenEmbryomics Pty. Ltd., Victoria, Australia; 4 Drug Delivery Disposition and Dynamics, Faculty of Pharmacy and Pharmaceutical Sciences, Parkville, Melbourne, Victoria, Australia; 5 Centre for Reproductive Health, Hudson Institute of Medical Research, Clayton, Victoria, Australia; 6 Department of Obstetrics and Gynaecology, Monash University, Clayton, Victoria, Australia; 7 Monash Women’s & Newborn Program, Monash Health, Victoria, Australia

**Keywords:** Genomics, Haplotypes

## Abstract

Whole-genome sequencing of preimplantation human embryos to detect and screen for genetic diseases is a technically challenging extension to preconception screening. Combining preconception genetic screening with preimplantation testing of human embryos facilitates the detection of de novo mutations and self-validates transmitted variant detection in both the reproductive couple and the embryo’s samples. Here we describe a trio testing workflow that involves whole-genome sequencing of amplified DNA from biopsied embryo trophectoderm cells and genomic DNA from both parents. Variant prediction software and annotation databases were used to assess variants of unknown significance and previously not described de novo variants in five single-gene preimplantation genetic testing couples and eleven of their embryos. Pathogenic variation, tandem repeat, copy number and structural variations were examined against variant calls for compound heterozygosity and predicted disease status was ascertained. Multiple trio testing showed complete concordance with known variants ascertained by single-nucleotide polymorphism array and uncovered de novo and transmitted pathogenic variants. This pilot study describes a method of whole-genome sequencing and analysis for embryo selection in high-risk couples to prevent early life fatal genetic conditions that adversely affect the quality of life of the individual and families.

## Introduction

### Whole-genome sequencing in the IVF clinic

For over two decades, preimplantation genetic testing (PGT) has been available for couples who are aware they carry a genetic condition or have had a child affected by a genetic disease. *In vitro* fertilisation (IVF) used in conjunction with monogenic PGT is available for couples to prevent transmission of known hereditary monogenic disorders. PGT for aneuploidy screens embryos for large segmental or whole-chromosome copy number changes and is commonly used for older women (>35 years) who have a history of infertility, miscarriages or chromosomally abnormal conceptions^1–3^. The most recent developments in clinical PGT are low-coverage next-generation sequencing and Karyomapping, which uses a highly polymorphic single-nucleotide polymorphism (SNP) microarray to identify disease-causing haplotypes. Next-generation sequencing PGT for aneuploidy (typically <0.1× depth) is useful for high-throughput screening at a reasonable cost for detecting chromosomal aneuploidies, structural variations and large copy-number variations (CNVs)^4–6^. In addition to pedigree analysis for monogenic disorders, Karyomapping has been reported to identify partial chromosomal aneuploidies as small as 1.8 Mb^7^.

For couples seeking to ascertain their risk of having an affected child, around 6,000 diseases exist that may be genetically screened for^8^. A mutation or disease-causing variant in one or both copies of approximately 5,000 human genes can cause a syndromic disease or phenotype^9–14^. Between 0.5–5% of infants are born with a genetic condition or disorder^15,16^. The preconception genetic screening panels that are available to determine a couple’s carrier status for disease-causing genetic variants are limited to a subset of high-risk genes^7^. Currently, preconception screening and PGT are performed as separate unlinked tests^17^. An estimated 74 de novo SNP mutations are introduced at embryogenesis, which, when expressed dominantly or as a compound heterozygote, result in severe pathogenic phenotypes^18–20^.

With the declining cost and increased availability of whole-genome sequencing, we sought to explore the design of combined preconception screening and embryo PGT using whole-genome sequencing to detect disease-causing genetic variants in couples and their embryos in accordance with recommended practice guidelines^21–23^. We investigated whether whole-genome sequencing of IVF-conceived embryos could screen for hereditary syndromic genetic diseases in addition to identifying the more technically challenging syndromes resulting from de novo mutations^10,15^. To date, whole-genome sequencing has been used in a limited number of assisted reproduction cases, principally due to the high cost of high-throughput sequencing^21^. We hypothesised that whole-genome sequencing of preimplantation embryos combined with sequencing genomic DNA from both parents could address the limitations associated with current PGT techniques. The aim was to use whole-genome sequencing analysis to screen embryos for pathogenic variants that would result in severe childhood-onset diseases^24^.

For this pilot study, we sequenced the genomes of five IVF couples and 11 of their IVF embryos that had previously undergone clinical PGT for familial diseases with Karyomapping^6,25^. The whole-genome amplified trophectoderm cell biopsy samples and the genomic DNA of the parents’ samples were used as template DNA for library generation for whole-genome sequencing. Each embryo’s resolved genome sequence was triangulated using multiple trio testing of their parents’ sequences for confirmation of variant status and vice versa^22^. To detect clinically actionable pathogenic variations, multiple trio testing of the parental and the embryo genomes was performed. This was followed by variant annotation using databases to grade variant pathogenicity and the use of pathogenicity prediction algorithms for inherited and de novo mutations and variants of unknown significance^26^. Detecting disease-causing pathogenic variants necessitated the use of inheritance-mode filtering to exclude false positives caused by sequencing artefacts. For each of the major modes of inheritance, curatable variant filter and classification sets were generated to detect known ClinVar archive pathogenic variants^27^. Variants of unknown significance were classified using a range of pathogenicity prediction algorithms and functional annotation databases. The threshold for classifying candidate pathogenic variants was based on pathogenic and likely pathogenic ClinVar categories^9^. For differentiating between type I and type II error calls for de novo mutations, we used the variant allele frequency (VAF) and quality by depth (QD) metric to filter false-positive pathogenic variants in combination. The purpose of this was to detect inherited pathogenic and unacceptably high-risk de novo variations that would be clinically actionable and to guide personalised diagnosis and treatment^28,29^. Our study to design and test a framework to determine clinically actionable pathogenic variants is, to our understanding, the first of its kind.

## Methods

### Study participants

Couples who had PGT for single gene disorders provided written informed consent to having whole-genome sequencing on themselves and their biopsied embryos included in the study^6^. Each participant was given the option to have the results of their genomic DNA and their biopsied embryo samples reported or withheld. All participating couples consented to whole-genome sequencing and elected to receive results for themselves and their tested PGT embryos. The study and protocol were approved by the Monash Health Human Research Ethics Committee (Ref: HREC/17/MonH/286) and all experiments were performed in accordance with protocol guidelines and regulations.

#### Library preparation and sequencing

Genomic DNA from five couples who had been used as reference templates for PGT using Karyomapping were selected for whole-genome sequencing. The DNA had been extracted from whole blood using a ReliaPrep™ Blood genomic DNA Miniprep System (Promega, USA). For the isolation of embryonic DNA, intracytoplasmic sperm injection method created embryos belonging to the five PGT couples underwent trophectoderm biopsy, using laser or mechanical techniques, on day five or six of culture to remove 4–10 trophectoderm cells. Biopsied cells were washed three times in a solution of 1× phosphate-buffered buffer (Cell Signalling Technologies, USA) and 1× polyvinylpyrrolidone (Cook Medical, Australia) followed by whole-genome amplification by multi-displacement amplification with SureMDA system (Illumina, USA) as per manufacturer’s instructions. Samples for whole-genome sequencing were selected based on Karyomapping quality control metrics, which indicated a SNP call-rate on the HumanCytoSNP-12 BeadArray of >96% and allele dropout and miscall rates of <1%. A 1 ug sample of parental genomic DNA and embryo whole-genome amplification products were sent to BGI Genomics (Tai Po, Hong Kong) for sequencing with the BGI-SEQ500. Briefly, the DNA samples were fragmented to approximately 350 bp with a E220 Covaris (Covaris Inc., USA) followed by 3′ end-repair, adaptor ligation and amplification by ligation-mediated polymerase chain reaction, single strand separation and cyclisation. DNA nanoballs were produced with rolling-circle amplification, placed in patterned nanoarrays which are 100 bp paired-end reads on a BGI-SEQ500^30^.

#### Read processing

Standard raw read processing through to variant call format was performed in accordance with Genome Analysis Toolkit best practices by the BGI Genomics Online portal pipeline^31,32^. Raw reads were mapped to the human reference genome (GRCh37/HG19) with Burrows-Wheeler Aligner^33,34^, polymerase chain reaction duplicates were removed using Picard tools^35^, local realignment was undertaken with Genome Analysis Toolkit^36,37^ and variants were called with HaplotypeCaller using the variant quality score recalibration method.

#### SNP and indel analysis

**Figure 1:**
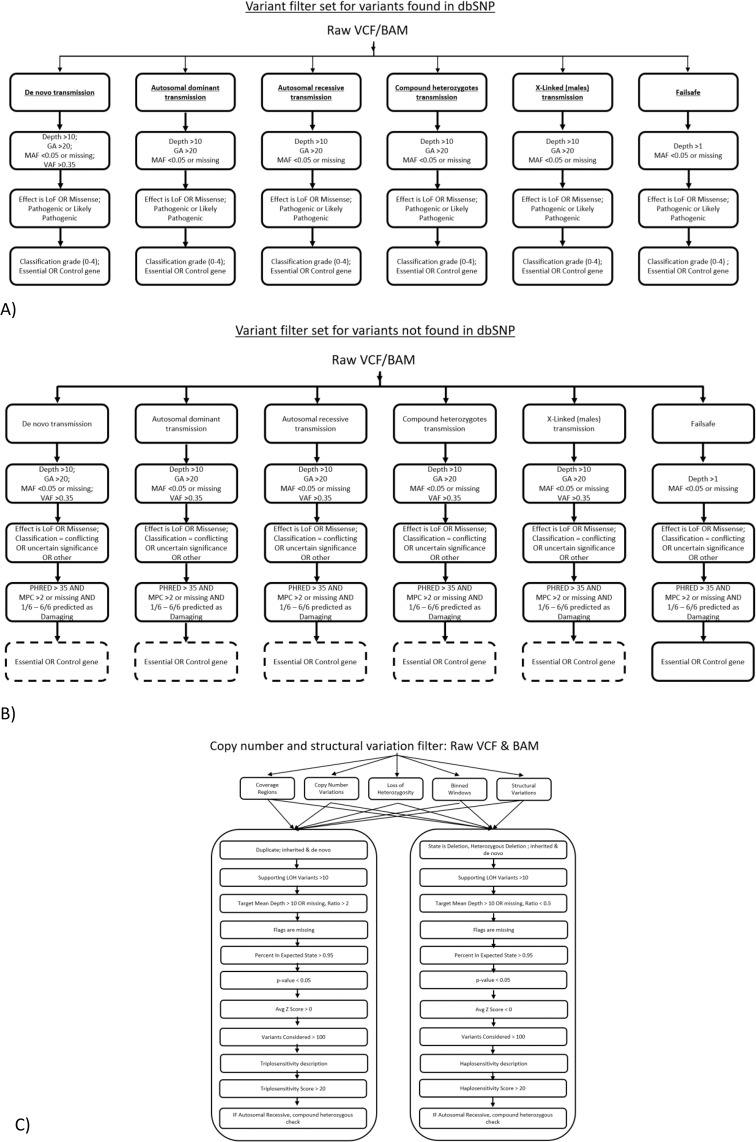
Filter sets for pathogenic variant detection from the classifications of variants: (**A**) variants classified as ‘likely pathogenic’ or ‘pathogenic’, (**B**) unclassified variants with a potentially feasibly damaging likelihood and (**C**) copy number variant calling pipelines.

Analysis was guided by the Standards and Guidelines from the American College of Medical Genetics for interpretation of sequence variants^38–40^. Clinically actionable variants were defined as those that could be justified in requesting for screening by an accredited medical ethics committee^41,42^. Each parental and embryo binary alignment map (BAM) and raw variant call format files were imported into VarSeq (GoldenHelix, USA). Variant filtering workflows were arranged for the inheritance modes of; dominant heterozygous, recessive homozygous, compound heterozygous, X-linked, de novo and a low-specificity high-sensitivity failsafe filter with a low depth threshold (read depth >1) and was missing the genotype quality filter (Supplementary Table 3). The failsafe filter therefore having intentionally high number of false positives for manual curation (Fig. 1). For variants of unknown significance or conflicting variants, a stringent pathogenicity functional prediction filter was set using the following prediction algorithms: SIFT, Polyphen2 HVAR, MutationTaster2, MutationAssessor, FATHMM and FATHMM MKL^43–47^. If more than one of the algorithms predicted a variant as damaging, the variant was retained. Variants were then filtered by MPC scores >2 and a final Phred-scaled CADD score of >35 concluded the mutation prediction filter set^48,49^. Short tandem repeats were calculated with ExpansionHunter version 2.5.5 using the default 17 tandem repeat loci to determine short tandem repeat numbers on embryos and parents^50^. Calculation was performed at BGI Genomics for the following loci provided by ExpansionHunter version 2.5.5: cbl proto-oncogene (*CBL*), atrophin 1 (*ATN1*), ataxin 2 (*ATXN2*), ataxin 3 (*ATXN3*), junctophilin 3 (*JPH3*), calcium channel, voltage-dependent, P/Q type, alpha 1A subunit (*CACNA1A*), dystrophia myotonica-protein kinase (*DMPK*), cystatin B (*CSTB*), *ataxin 10* (*ATXN10*), *ataxin 7* (*ATXN7*), huntingtin (*HTT*), protein phosphatase 2, regulatory subunit B beta (*PPP2R2B*), *ataxin 10* (*ATXN1*), chromosome 9 open reading frame 72 (*C9ORF72*), frataxin (*FXN*), androgen receptor (*AR*) and fragile X mental retardation 1 (*FMR1*) on all embryo and parental samples.

#### Copy number and structural variation

CNVs were called using CNVnator (v.0.2.7)^51^ and structural variations with Breakdancer^52^ and CREST^53^. A secondary, overlapping CNV discovery analysis was performed by binning into 10 kb windows, filtering by calling loss of heterozygosity (LoH) in more than 95% of variants in flagged regions^54,55^ and annotating using ClinGen Gene Dosage Sensitivity (27-09-2017 release). Structural variations were called and included in the analysis using Breakdancer^52^. CNVnator and Breakdancer calls were imported into Varseq and then compared with the inherited CNVs from each parent and categorised as having dosage pathogenicity for either haploinsufficiency or triplosensitivity. LoH regions (>100 and 95% of variants) were trio-called compared with the parental LoH regions. Filtering was applied for the haploinsufficiency and triplosensitivity categories of ‘sufficient evidence for dosage pathogenicity’ or ‘gene associated with autosomal recessive phenotype’ and called for pathogenicity using the target copy number state for proband per sample. This was performed by applying a ratio of >2.0 with a Z-score of >0 for duplications and <0.5 with a Z-score of <0, a mean targeted depth >5 and a lack of quality control flags (high control variation, low control depth, low Z-score or within regional interquartile range) for detecting true positive CNVs. CNVs with recessive inheritance were cross-checked against the autosomal recessive SNP and indel variants.

## Results

### PGT variant validation

Sequencing depth was comparable between the amplified trophectoderm-biopsy DNA from embryos and the parents’ from genomic DNA (mean depth of 48.2× versus 46.1×). Embryo reads were equivalent to the couple’s genomic DNA samples for raw and clean reads, bases aligned and transitions to transversion ratios of 2.071 and 2.081 (Supplementary Table 1 and Fig. 1a). Genome coverage for embryos and couples was comparable at sequencing depths of 4× and 10×. However at 20×, genome coverage was relatively decreased for biopsied embryos at 87.5% compared with 96.4% from genomic DNA (Supplementary Figs. 1b, 4a,b). Therefore, with the exception of the failsafe filter, variant filter sets each had the depth threshold at >10× coverage.

Assembly and mapping for the SNP and indel calls were highly concordant between embryos and couples (Supplementary Fig. 1c–f), except for novel SNPs, which averaged 85,527 (standard deviation [SD] 29,576.6) variants in embryos and 21,663 (SD 1102.4) variants for couples. This was reflected in the high number of LoH regions in embryos (5460, SD 1609 versus 3733, SD 87) that presumably indicates regions of allele dropout.

### De novo mutations

As expected for the couple’s male and female partners genomic DNA samples, non-homozygote VAFs showed a normal distribution, with the average centred at 0.5 (indicating 50% of reads per base, Supplementary Fig. 2b). The embryos heterozygote VAF distribution ranged from 0.08 to 0.34 with an average peak at 0.26 and maximum at 0.12 (Supplementary Fig. 2a). This low embryo VAF is believed to represent false positive heterozygote calls from either base misincorporation or read misalignment^22^. Due to this, the de novo filter included a false-positive filtering gate to remove de novo SNP variants with a VAF < 0.35, the rationale being that the failsafe filter will shortlist potentially dangerous or clinically actionable variants for individual curation. Variations involving deletions >1 bp had a higher VAF than those involving a base change, although we did not alter the filtering based on this as the upper limit was approximately consistent.

An additional quality by depth (QD) threshold of >12 was added to the non-dbSNP variant subfilters. This QD threshold reduced the number of de novo variants flagged for curation from 285 across all the eleven embryos to 57. QD filtering was not applied to the transmitted variants, but when this stringent filter was applied to the non-dbSNP variants, 8/125 unique and pathogenic transmitted variants were removed from reporting.

Variant filters were therefore arranged to classify for each mode of inheritance into two parallel sub-filter sets that all variants would be assessed; one sub-filter of each filter set for annotating variants catalogued in dbSNP and a second for variants not catalogued to date, for which pathogenicity prediction was used (Fig. 1a–c).

### Variant trio-calling

Three of the five couples had undergone PGT for autosomal dominant conditions, one for an autosomal recessive condition and one for an X-linked condition (Table 1). To confirm the embryo PGT results, in three of the five couples at least one euploid embryo was available (i.e. affected, carrier or unaffected). To determine the concordance between the whole-genome sequencing results to the HumanCytoSNP-12 BeadArray platform used for the couples clinical Karyomapping cycles, assessment of heterozygote calls (~75,000 variants) indicated >99.0% concordance with whole genome sequencing calls. Comparing the results of the pathogenic variants previously diagnosed during monogenic PGT cycles using Karyomapping to those obtained through whole-genome sequencing indicated complete concordance for both couples and embryos (Table 1). One embryo’s PGT variant had a substantially lower than expected VAF (0.143; 3/21 reads) but as this was a transmitted variant for it was called by the filter pipeline.

**Table 1:** Couples and embryo numbers by inheritance, disease status and type of variant.

PGT couple	PGT gene; disease (n = 10)	Inheritance	Embryo status (n = 11)	Variant
A	*PTPN11;* Noonan syndrome 1	Autosomal Dominant	1 x affected	SNP
B	*GLA;* Fabry disease	X-Linked recessive	1 x affected	SNP
C	*BRCA2;* multiple neoplasms	Autosomal Dominant	1 x affected, 1 x unaffected	Indel
D	*CFTR;* cystic fibrosis	Autosomal Recessive	1 x affected 1 x carrier 1 x unaffected	SNP
E	*KRT10;* epidermolytic hyperkeratosis	Autosomal Dominant	1 x affected 3 x unaffected	SNP

### Pathogenic and predicted pathogenic variant detection in embryos

**Figure 2:**
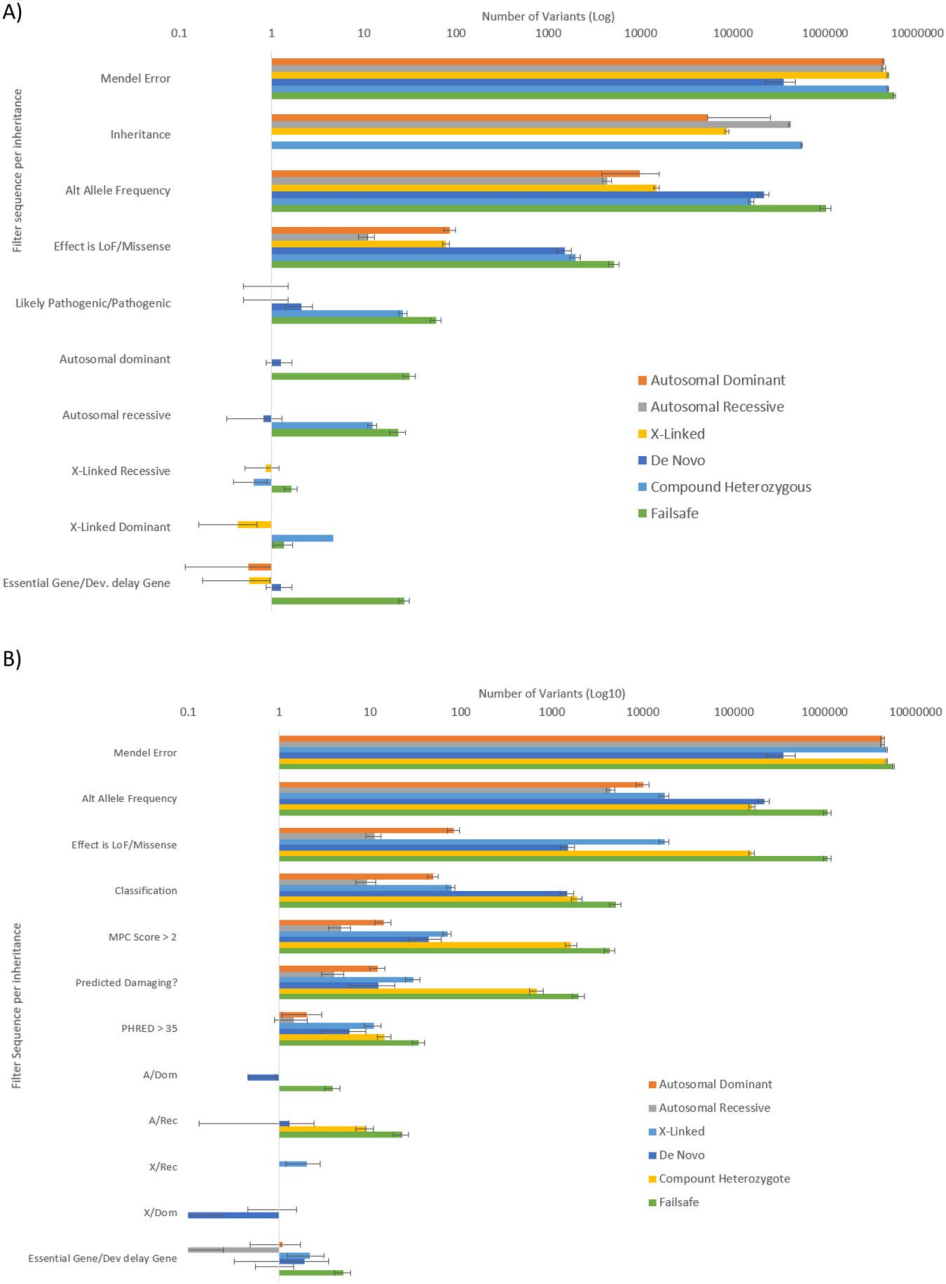
Bar graphs of the filter system for determining the clinically relevant variants proposed for embryo selection for each mode of inheritance: (**A**) filter sets for determining clinically relevant variants classified as either likely pathogenic or pathogenic and (**B**) filter sets for variants not yet classified but potentially damaging or disease causative. Filters in each row are successively added to the total number of variants remaining.

For the recessive filter there was an average of 0.82 transmitted pathogenic variants found in dbSNP per embryo (build 151, ranging between 1 and 2 stars for ClinVar review status, 0 stars representing no assertion criteria or minimal evidence, up to 4 stars for clinical practice guideline). This is compared to an average of 1.27 non-inherited variants per embryo that were predicted pathogenic (Fig. 2, excluding variants for which the couples had originally sought PGT). In one of the couples, both were heterozygote carriers of the *CTFR* ΔF508 mutation and resulted in a heterozygote in at least one embryo.

For the dominant filters, 1.27 pathogenic variants per embryo were in dbSNP, compared to a mean of 0.45 non-dbSNP predicted pathogenic variants. To detect transmitted pathogenic or predicted pathogenic variants occurring in regions of allele dropout and/or low-coverage in the amplified embryo DNA compared to parental sequences that used genomic DNA, LoH was used (>95% and 100 variants) for variants which had fewer than 10 reads. An average sum of 2.3 (SD 1.2) pathogenic or predicted pathogenic variants were noted as expected but missing from the embryo sequencing due to low coverage threshold or LoH from all the filters. Pathogenic variants in low-coverage regions were phased using the nearest flanking SNPs of the missing regions to determine the carrier status. A mean of 4.5 (SD 3.7) likely pathogenic or pathogenic variants were found in embryos and a mean of 5.5 (SD 3.4) variants deemed potentially pathogenic and required haplotype curation via LoH to account for dropout of potentially inherited but missing pathogenic variants.

To prevent filtering of true positive de novo mutations, the failsafe filter container was used to capture clinically relevant variants for curation. After elimination of PGT variants, 17 variants were detected in the 11 embryos with review status of 3 stars, of which none were clinically actionable essential or developmental delay genes and were removed following QD filtering. Review status classification revealed that only the failsafe filters had missing calls, with a mean of 2.36 (SD 3.86); none of the variants captured by the failsafe filter resulted in compound heterozygotes derived from transmitted variants. There were no ClinVar review status 1-star (conflicting interpretations) variants found in any of the embryo samples. Similarly, there were no compound heterozygotes, homozygous autosomal recessive or X-linked (in females), or likely pathogenic or pathogenic in American College of Medical Genetics incidental findings variants in embryos or parental genomes. There were 109 unclassified candidate pathogenic de novo mutations across the 11 embryos with nine variants featured repeatedly across multiple embryos, all but two of which occurred in more than one family. There were 10 candidate de novo autosomal dominant variants in four embryos which had a VAF < 0.4 and only one having a VAF > 0.5, indicating the high likelihood of false-positive calls. Addition of the QD minimum threshold to the unclassified filters for QD < 12 reduced the candidate false positive unclassified variant calls to one de novo mutation at the *ABL1* locus (rs121913459, VAF 0.63, QD = 20.9) in a single embryo^56^.

### Tandem repeat disease loci analysis

For the 17 loci that Expansion Hunter assessed the tandem repeat number at known disease-causing loci, no parental samples indicated pathogenic repeat numbers. In embryo samples, most of the loci tested provided at least one concordant call in terms of transmission exactness. At three loci, both alleles were discordant: *FMR1*, *ATXN1* and *ATXN3*.

### Copy-number and structural variations

**Figure 3:**
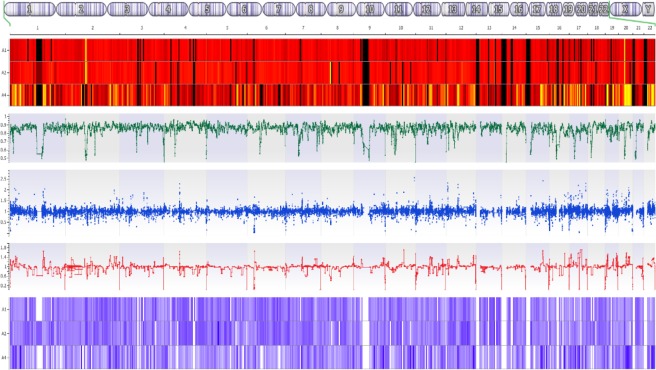
Copy number variant charts for an embryo genome sequencing sample from chromosomes 1–22: (**A**) Target mean depth, where the top intensity bar is the paternal depth, the central bar is the maternal depth and the lower bar is the embryo depth (black indicates no coverage and yellow indicates high coverage); (**B**) loss of heterozygosity proportion of the variants in the expected state of variant heterozygosity loss for the embryo (green dots); (**C**) ratio of coverage regions for the embryo sample (blue connector); (**D**) ratio of binned regions in 10 kb windows (red connector). (**E**) z-scores of the parents and embryo samples, where the top intensity bar is the paternal depth, the central bar is the maternal depth and the lower bar is the embryo depth (light purple indicates a low a-score dark purple indicates a high z-score).

CNVs were assessed by direct transmission and binning reads in 10 kb windows and comparing against inheritance and ClinGen dosage sensitivity scores for pathogenicity. CNVs calls were higher in the embryos compared to parental samples, except for inter-chromosomal structural variants and structural deletions, suggesting a high false-positive rate (Supplementary Fig. 1f and Supplementary Table 1). As anticipated from the Karyomapping results, no pathogenic CNVs were detected (Fig. 3). There was a mean of 2.0 deleterious autosomal recessive structural variations for both couples and embryos compared with a mean of 5.21 and 8.05 structural variations for couples and embryos, respectively, for which triplosensitivity was contributing as autosomal recessive.

## Discussion

The purpose of this study was to develop a method of whole-genome sequencing analysis that could be used to screen human embryos for pathogenic variants. To achieve this, we firstly used parental genome sequences to identify the transmitted variants. Embryo biopsy samples that had undergone multiple displacement amplification and parental genomic DNA samples obtained from blood were used as templates for generating DNA libraries that were subsequently sequenced. Sequenced genomes of embryos and parents were analysed using variant annotation databases and functional prediction algorithms to detect the transmission or introduction of pathogenic mutations. Parallel filter sets were arranged to filter separately to predict unacceptably high-risk or known pathogenic variations, CNVs or chromosomal scale rearrangements. Multiple trio-testing of each embryo against the couples’ genomes facilitated the detection of transmitted and de novo variants calling as likely pathogenic or pathogenic by disorder or variant categorisation. The complete concordance between variant calls on the SNP array and whole-genome sequencing results indicated that inherited variants were confidently detected via trio-testing.

De novo variant calling in embryos presented a unique challenge. A custom VAF filter was required to minimise false positives that were likely introduced as a result of multiple displacement amplification from single base substitutions. The VAF soft threshold of <0.35 and quality scores guided the de novo variant calling. This threshold was marginally higher than the reported de novo false-positive threshold of 0.28 to 0.33^22^. We used VAF, base quality metrics and functional interpretation to determine pathogenicity to differentiate between true- and false-positive calls. Strict filtering of de novo mutations and the risk of under-calling was offset by the failsafe filter set, which was intended to perform a low-sensitivity function that would pick-up clinically actionable variants. Individual curation of these candidate variants indicated that these were likely to be false positives based on low VAF. To validate specific de novo variants, performing direct polymerase chain reaction following embryo re-biopsy or from DNA obtained from culture media are feasible options^57^. The known PGT variant occurring at an extraordinarily low VAF (0.143) in one of the embryos exemplifies the necessity to have specific filter sets for each mode of transmission and variant subtype.

To avoid pathogenic variants being transmitted in low or missing coverage regions and being undetected, an untransmitted variant filter manually examined uncalled variants flanking haplotypes to confirm the result at each site. The uniform coverage exhibited by multiple displacement amplification of DNA from the embryos suggests that the likelihood of a pathogenic de novo mutation arising in a region with low coverage is remote. These type 2 errors are further mitigated by the failsafe low-coverage assessment filter, although LoH and VAF filtering can guide manual decision-making. We avoided imputation for regions of LoH to focus on what could be ascertained directly from the data.

For this study we performed pathogenic variant detection of known likely pathogenic and pathogenic variants in accordance with available databases of variants that have high to complete penetrance. Further work is required to stratify the outcomes of compound heterozygotes in which at least one variant is ranked likely pathogenic. Here, we used a non-exhaustive list of essential genes combined with known developmental delay genes. A list of core disease genes for embryo genome screening is necessary to avoid overcalling^58^.

For CNV calls, the recommended 10 kb size for the bins represents the lower limit for the annotation software, which coincides with the upper limit for variant call format file indels. For variations exceeding 10 kb, variant calls were inconsistent between the couples and the embryos, and a read-binning approach was required to confidently call CNV and structural variations. CNV detection via analysis of 10 kb bins overcomes the issue of high false-positive CNV calls, as evidenced by the concordance between partner and embryo genomes. The effective 10 kb upper size limit of indels is conveniently bridged by performing binned CNV analysis in 10 kb blocks. This addressed the issue of the limitations of multiple displacement amplification, enabling comprehensive compound CNV detection of inherited variants and de novo mutations. Short tandem repeat loci yielded inconsistent results for parental and embryo genomes, an observation not pursued further. Clinically, it would be beneficial to use preconception short tandem repeat assessment of premutations at loci responsible for short tandem repeat disorders.

There are limitations to this pilot study and areas where further work is required. Pathogenic de novo mutations occurring in a region of no or low coverage will be a challenging limitation to overcome. Further work is required to determine the likelihood of one of these highly improbable scenarios occurring. A second limitation is the threshold of VAF, which obfuscates de novo mutation calling. The need to determine the validity of de novo mutation calls meant filtering out variants which were likely polymerase base incorporation errors of the MDA, allele dropout or mis-aligned reads, generating false-positive variants. The advantages to embryo development and implantation rates conferred by the technique of trophectoderm biopsy of 4–8 cells serves as an additional benefit by maximising embryo genome sequencing coverage. Although the VAF suggested that the type of mutation varies in mean VAF, this was not explored in the present study. Minimising amplification and sequencing artefacts through allelic ratio and haplotype scoring effectively minimises the number of candidate de novo mutations to a number that can be, if necessary, curated. An ethnicity-specific penetrance magnitude metric to guide the level of pathogenicity would be highly relevant for IVF-based screening.

Controversy regarding whole-genome sequencing in IVF is reflected in contemporary questions of the utility of transferring chromosomally mosaic embryos in PGT aneuploidy screening. We provide compelling evidence in favour of using whole-genome sequencing for screening embryos for pathogenic, severe disease-causing and unacceptably high-risk de novo mutations. Offering clinical genome screening of embryos in the IVF clinic, either as a standalone test or after low-coverage PGT, is based on evidence that the major classes of pathogenic variation can be reliably detected. In addition to comprehensive genomic screening, several embryo development-related aneuploidies, that cannot currently be screened for via next-generation sequencing based PGT (i.e. 69XXX and low-level mosaicism), can be directly observed and screened via this protocol because of its unlimited resolution of structural variation. Although low-coverage PGT for aneuploidy is effective for detecting large (>10 Mbp) chromosomal aneuploidies, 1–2% of conceptions carry a de novo CNV or structural aneuploidy of >100 kb, a significant gap in the detection threshold^16^.

The concept of applying whole-genome sequencing for PGT is contentious, the main concern being the sensitivity and specificity of a testing system and the ethical questions that arise^59–62^. The ongoing emotional and psychological burden born by the parents and the monetary cost of support from a healthcare system for caring for an affected individual is vastly greater than the cost of a genome sequencing test^63^. For IVF patients, undiagnosed reasons for a couple’s subfertility can be diagnosed and factored into the initial screening to produce a viable pregnancy. Additionally, pharmacogenetics guided stimulation regimens for oocyte retrieval and personalised embryo culture media based on metabomic pathway analysis could be ascertained.

The method we propose for screening embryos for pathogenic content has provided evidence of the feasibility of whole-genome sequencing to screen biopsied IVF embryos for severe disease-causing pathogenic variants. By including de novo mutations and premutation short tandem repeat disorders in preconception testing, the risk of childhood disease with known genetic aetiologies can be significantly reduced, should any couple choose to. The discovery of the *CFTR* ΔF508 mutation in one of the couples having PGT for an alternative mutation exemplifies the justification, relevance and utility of this study.

This study is the first to demonstrate the validity of using whole-genome sequencing in the IVF clinic. Further research is required for stratifying variant penetrance across ethnicities and expanding the variant data to include variants of unknown significance and idiopathic disorders with polygenic risk is warranted.

## Supplementary information

Supplementary Information.
