# Supplementary material for: Genome sequencing of human in vitro fertilisation embryos for pathogenic variation screening
Source: Sci Rep. 2020 Mar 2;10:3795. doi: 10.1038/s41598-020-60704-0 (PMC7052235; doi:10.1038/s41598-020-60704-0)
Supplement: Supplementary file 1 — Supplementary Information. [file 41598_2020_60704_MOESM1_ESM.docx]

**Supplementary Information**

**Genome sequencing of human in vitro fertilisation embryos for pathogenic variation screening**

*Nicholas M. Murphy^1,2,3,4^, Tanya S. Samarasekera^2^, Lisa Macaskill^2^, Jayne Mullen^2^, Luk J.F. Rombauts^2,5,6,7^

1. Genetic Technologies Ltd., Victoria, Australia
2. Monash IVF, Clayton, Victoria, Australia
3. GenEmbryomics Pty. Ltd., Victoria, Australia
4. Drug Delivery Disposition and Dynamics, Faculty of Pharmacy and Pharmaceutical Sciences, Parkville, Melbourne, Victoria, Australia
5. Centre for Reproductive Health, Hudson Institute of Medical Research, Clayton, Victoria, Australia
6. Department of Obstetrics and Gynaecology, Monash University, Clayton, Victoria, Australia.
7. Monash Women’s & Newborn Program, Monash Health, Victoria, Australia

Correspondence:

Dr Nicholas Mark Murphy

[Nicholas.murphy@monash.edu](mailto:Nicholas.murphy@monash.edu)

Supplementary Figures

**Supplementary Figure 1a:** Whole genome sequencing quality control of DNA from amplified trophectoderm biopsied embryo samples genomes (blue) to DNA extracted from whole blood (green).


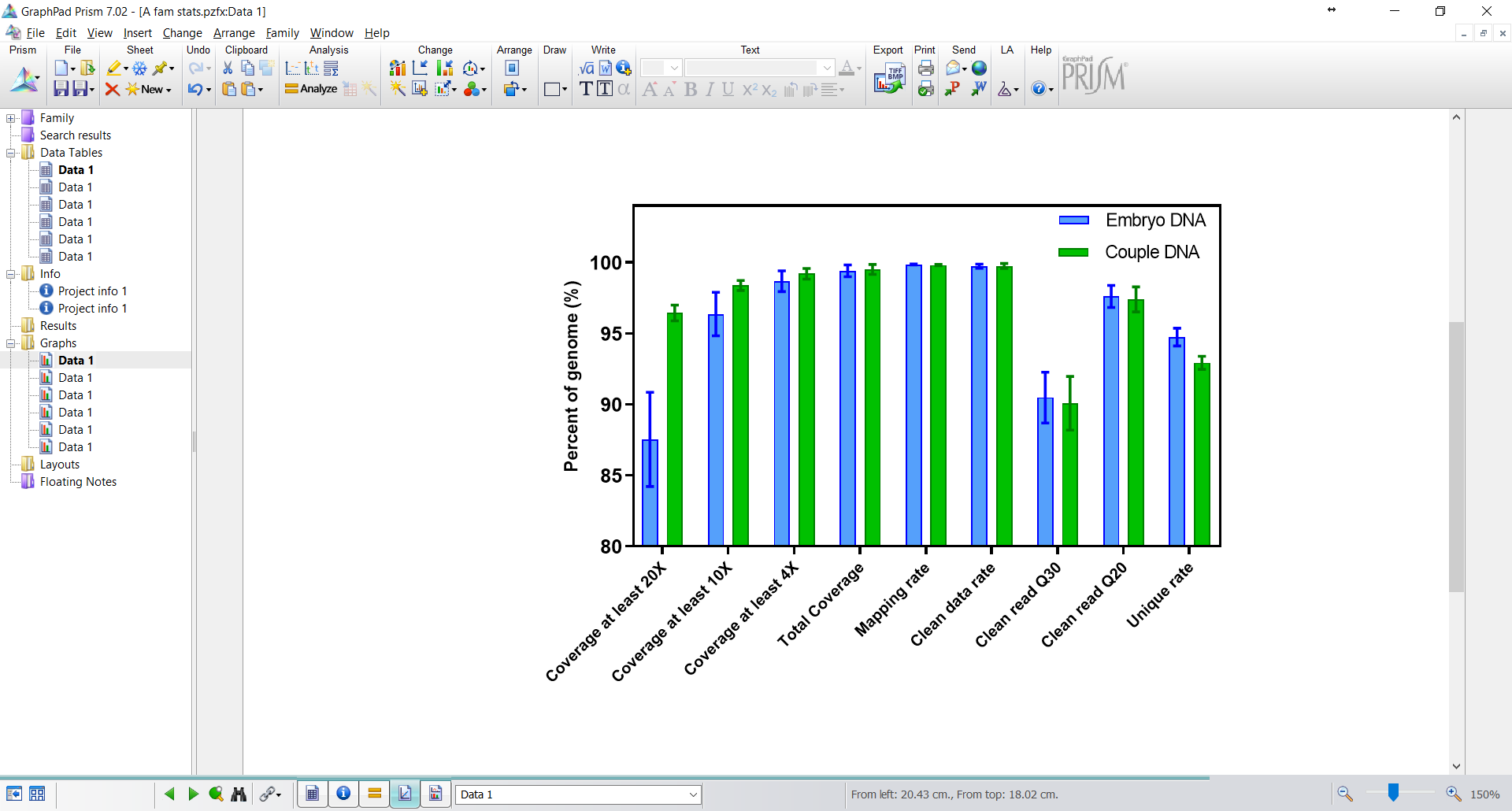


**Supplementary Figure 1b:** Results of mapping of whole genome sequencing of DNA from amplified trophectoderm biopsied embryo samples genomes (blue) to DNA extracted from whole blood (green). Coverage metrics describe the percentage of the genome that had the indicated level of coverage.


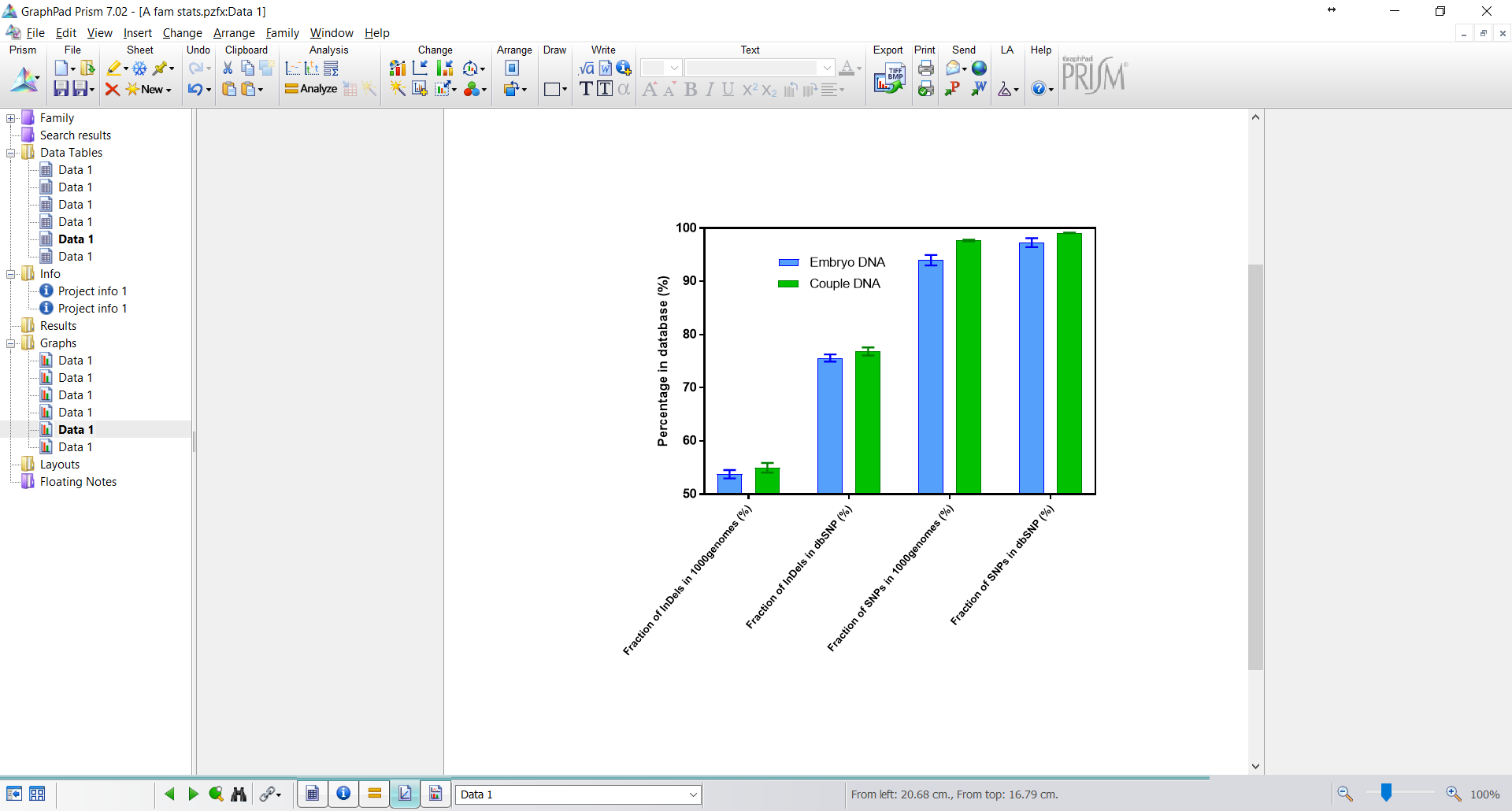


**Supplementary Figure 1c:** Unfiltered SNP and Indel results from databases recognising conserved human variants from whole genome sequencing of DNA from amplified trophectoderm biopsied embryo samples genomes (blue) to DNA extracted from whole blood (green).


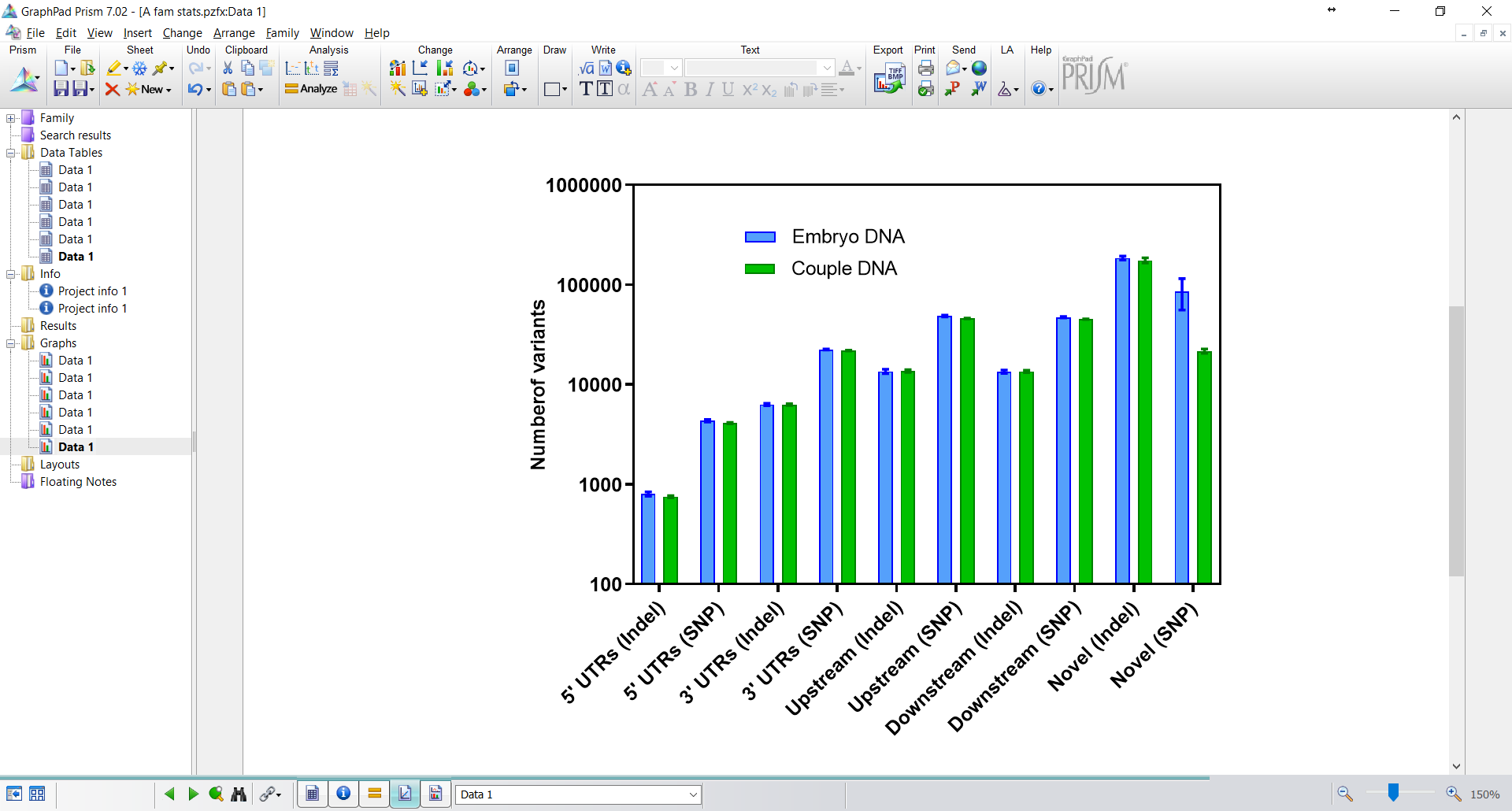


**Supplementary Figure 1d:** SNP variant differences between embryos and couple DNA whole genome sequences, upstream and downstream of coding regions for SNPs and Indels.


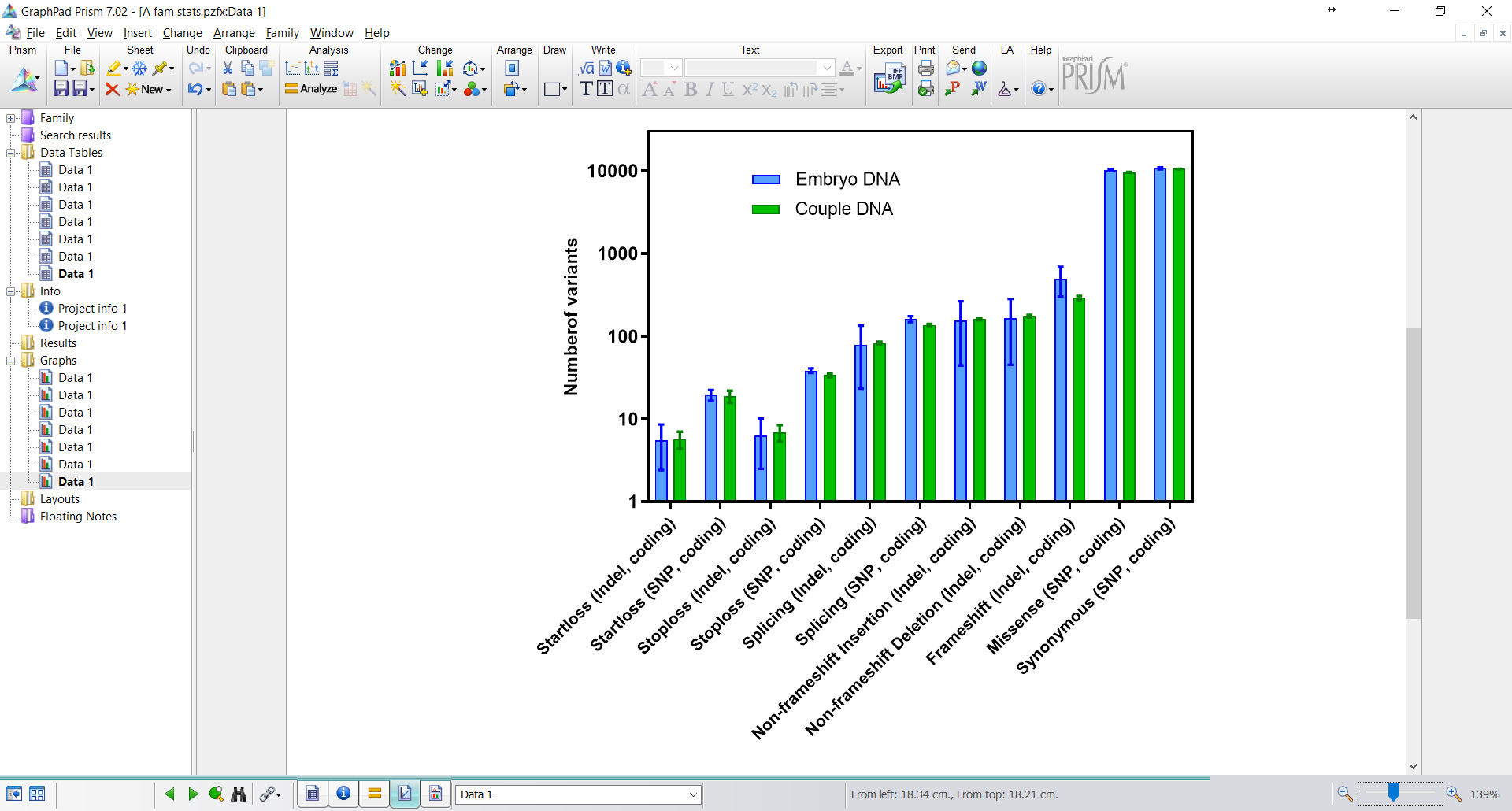


**Supplementary Figure 1e:** Variant subtype metrics for coding regions of embryos and couple genome sequences.

**Supplementary Figure 1f:** Copy number variation (CNV) and structural variation (SV) metrics between embryo and couple genome sequences.

a)


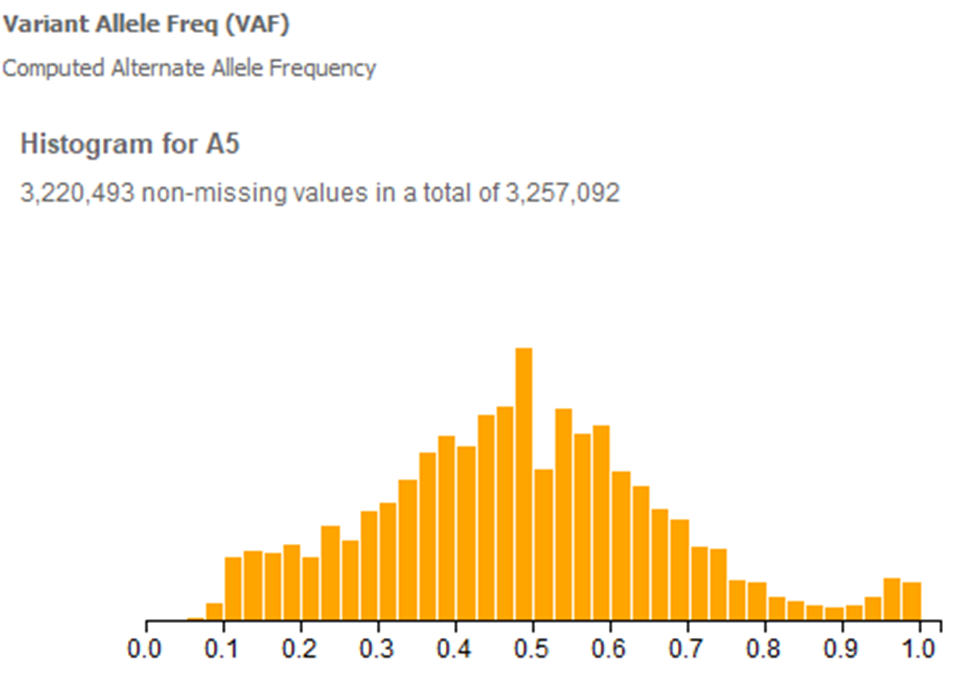

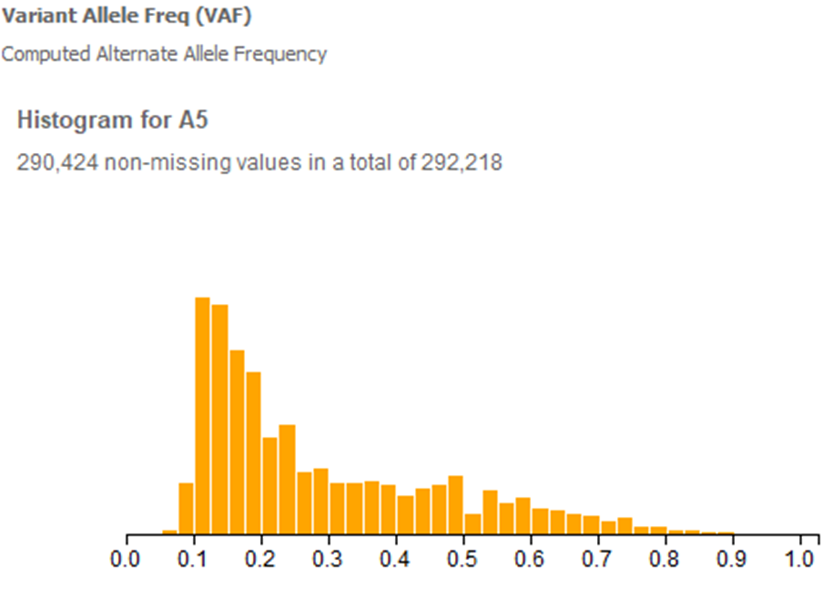

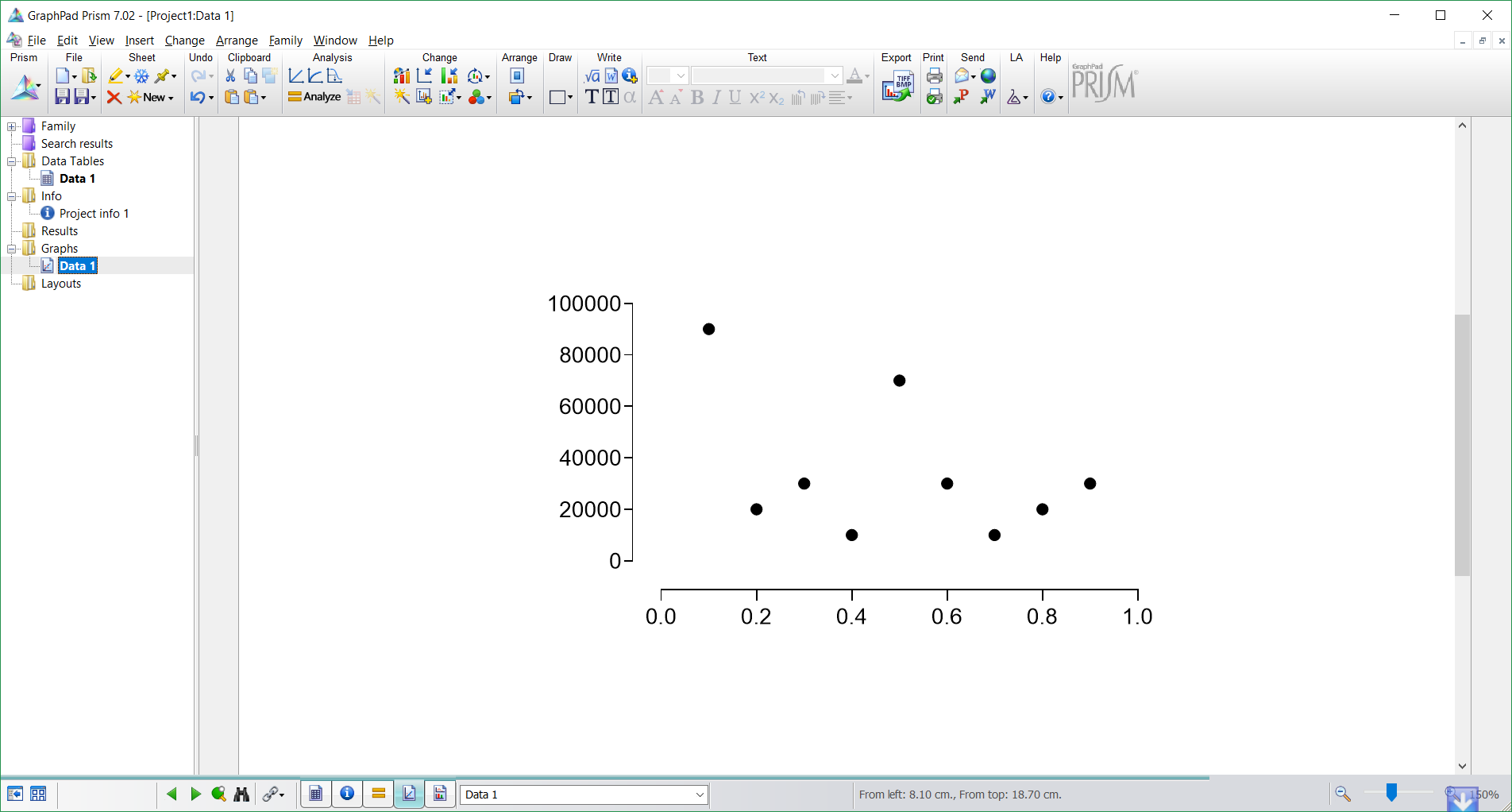

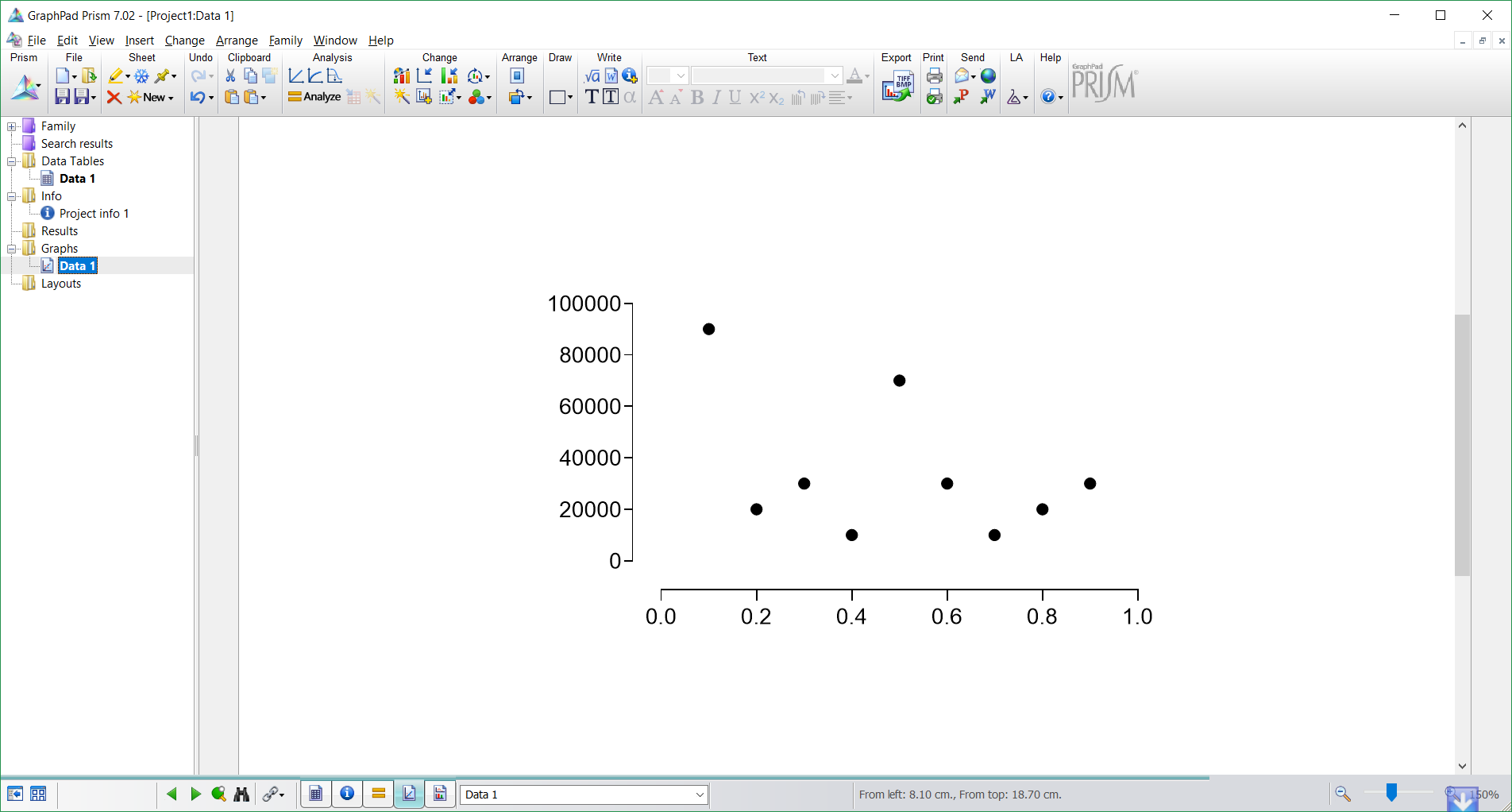

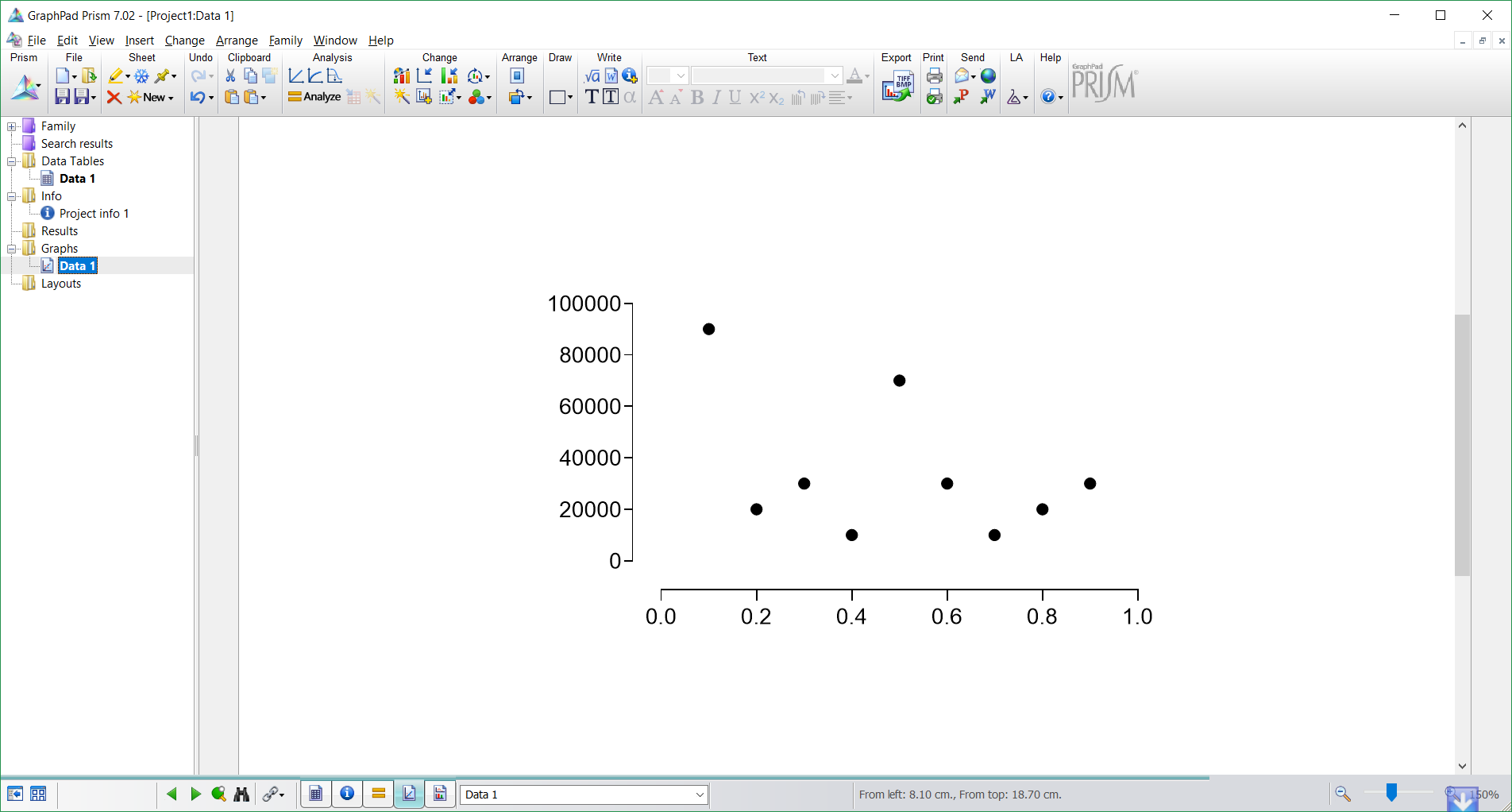

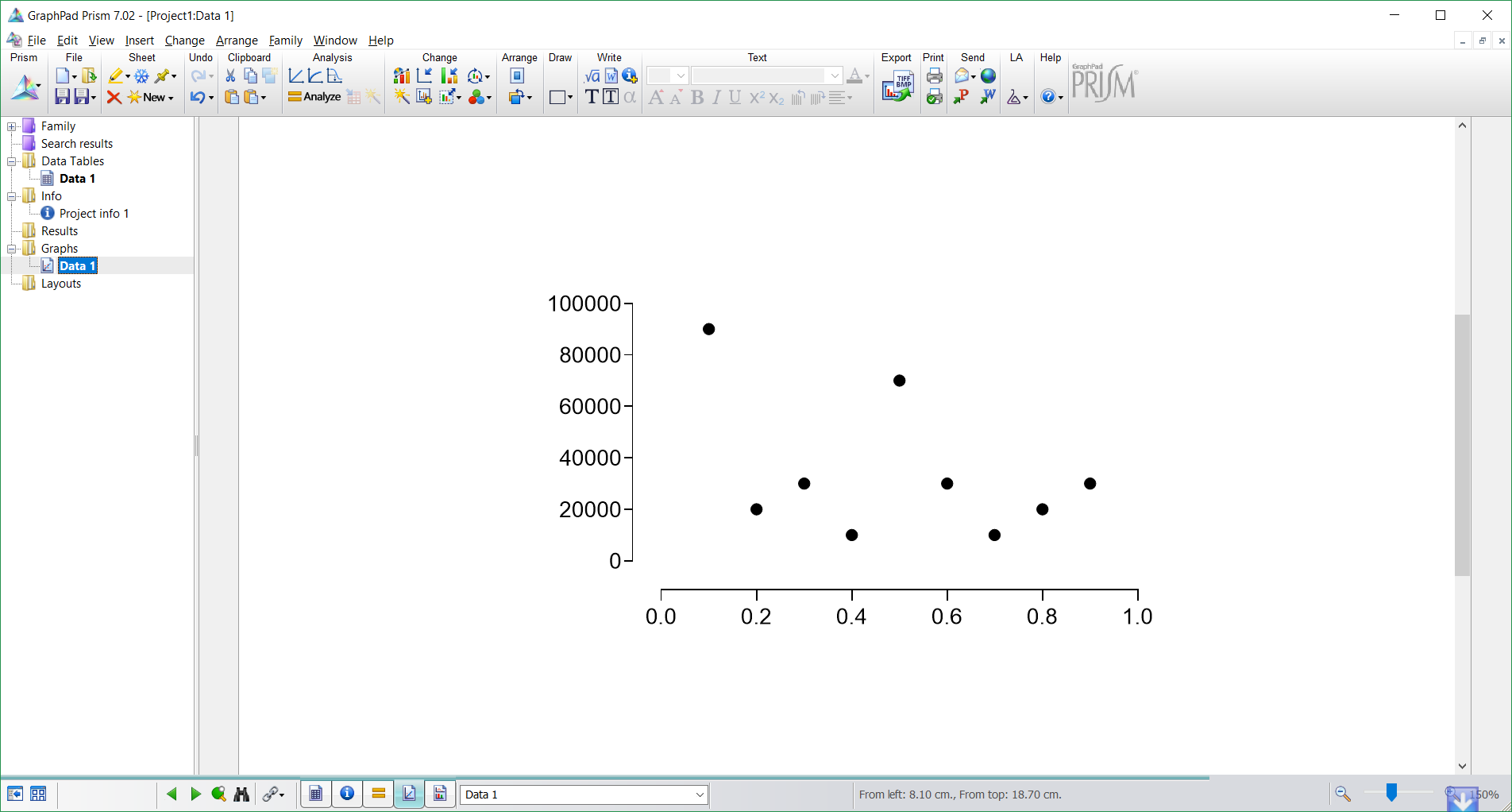

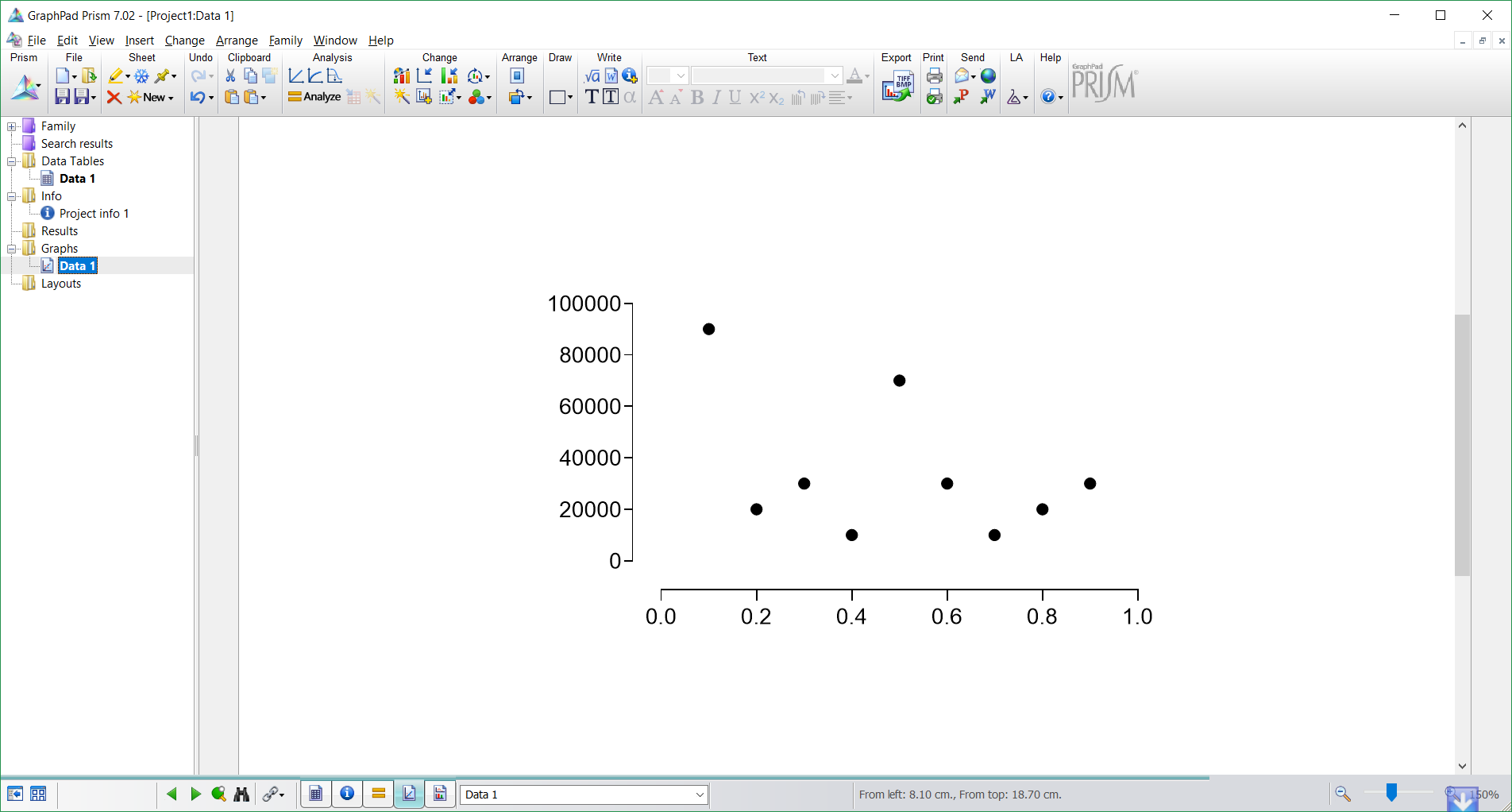


Number of variants

b)

A)


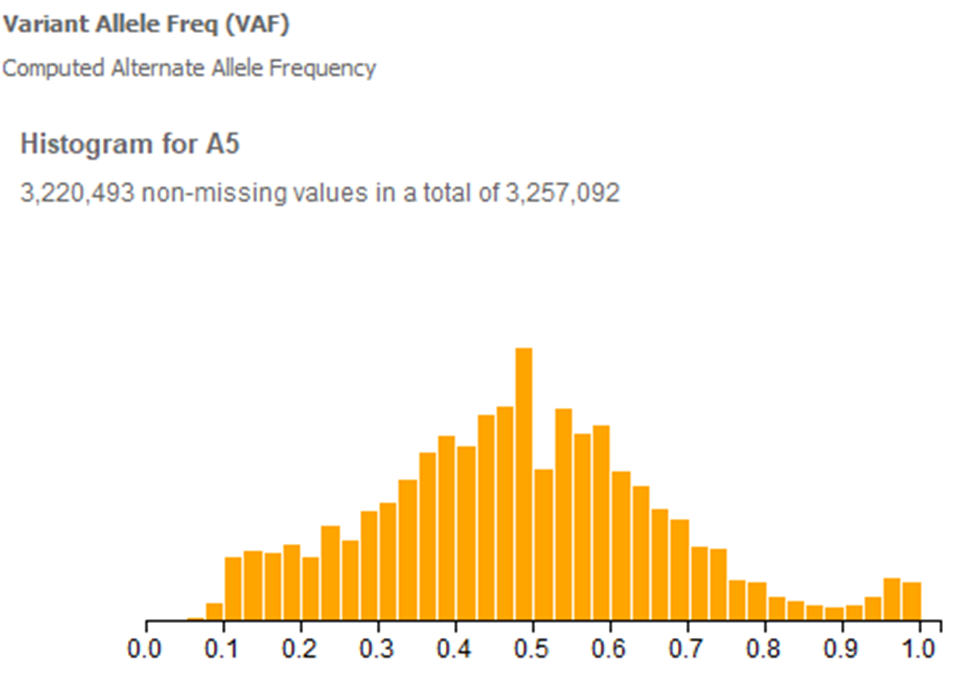

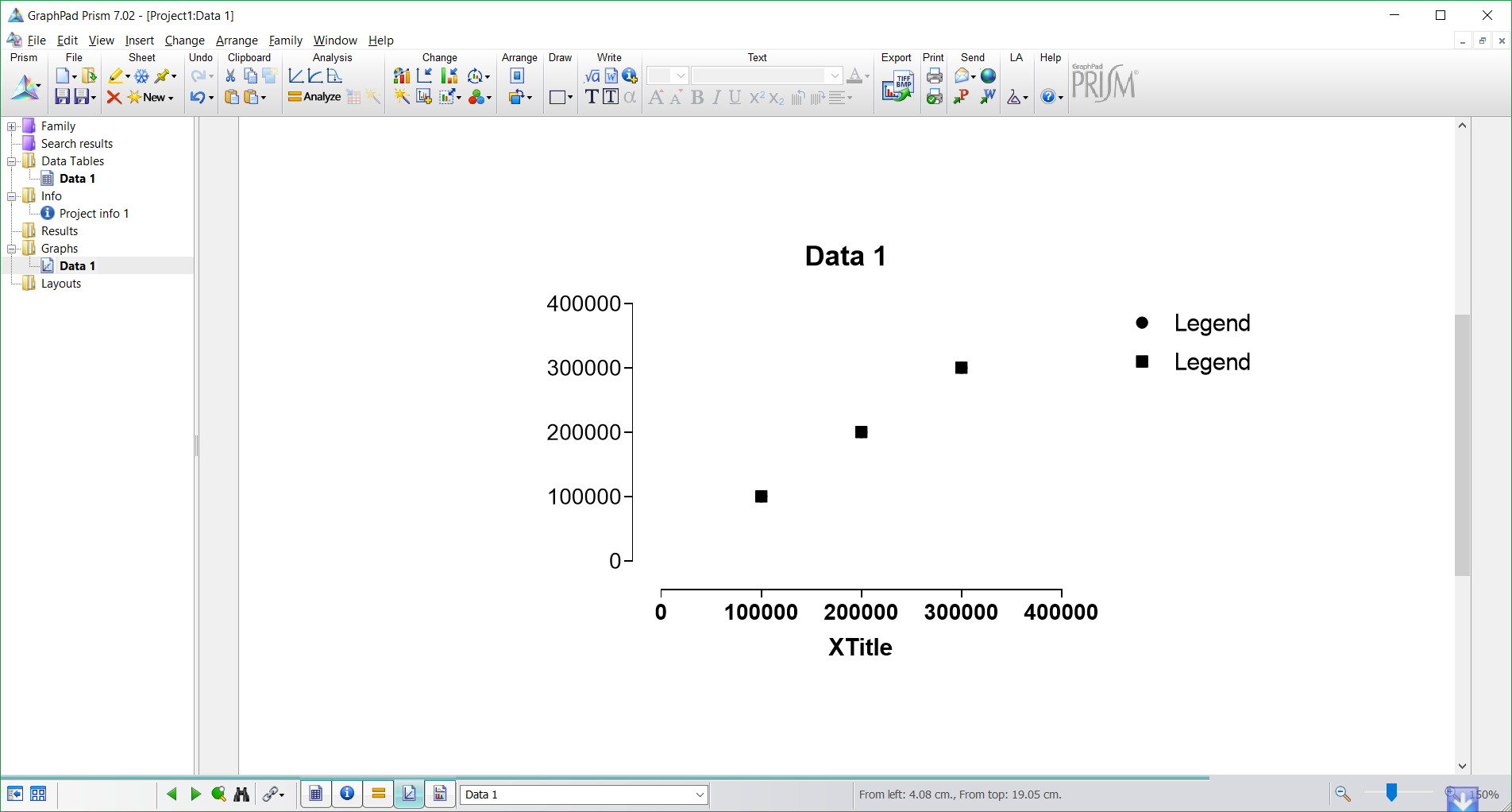

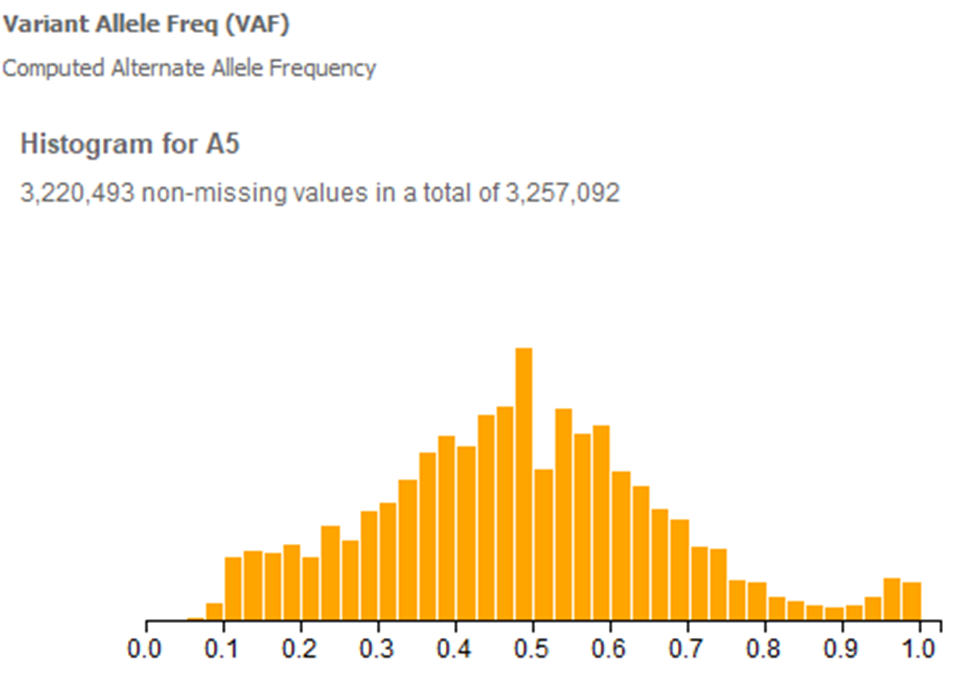


Number of variants

B)

**Supplementary Figure 2:**

a) Variant allele frequency (VAF) for candidate de novo variants from a typical embryo in windows of VAF 0.025 prior to filtering QD <12.

b) Variant allele frequency (VAF) for candidate de novo variants from a typical parent in windows of VAF 0.025.

**Supplementary Tables**

**Supplementary Table 1**

|  | **Couple** | **SD.** | **Embryo** | **SD.** |
| --- | --- | --- | --- | --- |
| **Total SNPs** | 3434300.5 | 2654.5 | 3581783.3 | 20036.6 |
| **Fraction of SNPs in dbSNP (%)** | 99.1 | 0.0 | 97.5 | 0.6 |
| **Fraction of SNPs in 1000genomes (%)** | 97.6 | 0.2 | 94.2 | 0.7 |
| **Novel** | 22487.5 | 710.5 | 78034.3 | 20426.6 |
| **Homozygous** | 1400593.0 | 34778.0 | 1425083.0 | 6921.2 |
| **Heterozygous** | 2033707.5 | 32123.5 | 2156700.3 | 24601.5 |
| **Intronic** | 1353220.5 | 2800.5 | 1395884.3 | 9904.3 |
| **5' UTRs** | 4122.5 | 45.5 | 4416.8 | 49.6 |
| **3' UTRs** | 22034.5 | 95.5 | 22576.5 | 138.9 |
| **Upstream** | 46326.0 | 141.0 | 48901.5 | 228.7 |
| **Downstream** | 45418.5 | 146.5 | 47617.5 | 311.2 |
| **Intergenic** | 1934455.0 | 717.0 | 2032045.8 | 10056.2 |
| **Ti/Tv** | 2.1 | 0.0 | 2.1 | 0.0 |
|  |  |  |  |  |
| **Total InDels** | 859161.0 | 17947.0 | 860210.3 | 17020.4 |
| **Fraction of InDels in dbSNP (%)** | 75.8 | 0.6 | 74.9 | 0.4 |
| **Fraction of InDels in 1000genomes (%)** | 53.6 | 0.6 | 52.6 | 0.5 |
| **Novel** | 190574.5 | 8988.5 | 198125.3 | 7305.3 |
| **Homozygous** | 285024.5 | 5710.5 | 284975.5 | 2923.9 |
| **Heterozygous** | 574136.5 | 23657.5 | 575234.8 | 19507.1 |
| **Intronic** | 363761.0 | 6904.0 | 364131.5 | 6674.4 |
| **5' UTRs** | 745.0 | 30.0 | 805.8 | 14.7 |
| **3' UTRs** | 6383.0 | 117.0 | 6485.3 | 87.0 |
| **Upstream** | 14015.0 | 315.0 | 14271.8 | 131.0 |
| **Downstream** | 13942.0 | 302.0 | 14051.0 | 150.9 |
| **Intergenic** | 458209.5 | 10244.5 | 458179.0 | 9990.6 |

**Supplementary Table 2:** Autosomal dominant de novo mutations identified in the biopsied embryos.

| **Gene** | **Disorders** | **Chromosome: Position; (rsID)** | **Variant Allele Frequency** |
| --- | --- | --- | --- |
| Lamin A/C (*LMNA*) | Charcot-Marie-Tooth disease, type 2 Muscular Dystrophy, Congenital, Lmna-Related | 1:156096660; (rs1048086299) | 0.37 |
| Ryanodine Receptor 2 (*RYR2*) | Ventricular Tachycardia, Catecholaminergic Polymorphic, 1, With Or Without Atrial Dysfunction And/Or Dilated Cardiomyopathy and Arrhythmogenic Right Ventricular Dysplasia, Familial, 2. | 1:237942068; (rs754610647) | 0.42 |
| Zinc Finger Protein 644 (*ZNF644*) | Myopia 21 | 1:91405028; (rs945688793) | 0.38 |
| Chromodomain Helicase DNA Binding Protein 4 (*CHD4*) | Sifrim-Hitz-Weiss Syndrome and Cellular Schwannoma | 12:6682336 | 0.44 |
| Titin (*TTN*) | Hereditary Myopathy With Early Respiratory Failure and Tibial Muscular Dystrophy, Tardive | 2:179541988 | 0.36 |
| Peroxisome Proliferator-Activated Receptor Gamma, Coactivator 1 Beta (*PPARGC1B*) | Body Mass Index Quantitative Trait Locus 11 and Kidney Lipoma | 5:149212261; (rs773389723) | 0.37 |
| Proto-Oncogene Tyrosine-Protein Kinase/V-Abl Abelson Murine Leukemia Viral Oncogene Homolog 1 (*ABL1*) | Congenital heart defects and skeletal malformations syndrome (CHDSKM) | 9:133748283 (rs121913459) | 0.63 |
| Collagen Type V Alpha 1 Chain (*COL5A1*) | Ehlers-Danlos Syndrome, Classic Type | 9:137703371 | 0.40 |
| Transcription Factor Binding To IGHM Enhancer 3 (*TFE3*) | Renal Cell Carcinoma, Xp11-Associated | X:48888094 | 0.40 |
| Patched 1 (PTCH1) | Basal Cell Nevus Syndrome and Holoprosencephaly 7 | 9:98270530 (rs1057518664 ) | 0.38 |
| Catechol-O-Methyltransferase (COMT) | Schizophrenia and Panic Disorder 1 | 22:19950266 | 0.41 |
| Dynein Cytoplasmic 1 Heavy Chain 1 (DYNC1H1) | Spinal Muscular Atrophy, Lower Extremity-Predominant, 1, Autosomal Dominant and Charcot-Marie-Tooth Disease, Axonal, Type 2O. | 14:102452243 | 0.36 |
| Pre-B-Cell Leukemia Transcription Factor 1 (PBX1) | Congenital Anomalies Of Kidney And Urinary Tract Syndrome With Or Without Hearing Loss, Abnormal Ears, Or Developmental Delay and Leukemia, Acute Lymphoblastic 3 | 1:164781366 | 0.38 |

**Supplementary Table 3:** Variant filter container arrangement

| De Novo Filter Container |
| --- |
| (Read Depths (DP) (Current) > 10 AND Genotype Qualities (GQ) (Current) > 20 AND Mendel Error (Current) is de Novo Allele AND (Alt Allele Freq (AF) < 0.05 OR missing) AND (Effect (Combined) is (LoF, Missense)) AND Sequence Ontology (Combined) AND Quality by Depth (QD) > 12 AND (Variant Allele Freq (Current) >= 0.35 OR missing) AND (Classification is (Likely Pathogenic,  Pathogenic)) AND Review Status AND (A/Dom genes is true OR A/Rec genes is true OR X/Rec is |

| true OR X/Dom is true)) OR (Read Depths (DP) (Current) > 10 AND Genotype Qualities (GQ) (Current) > 20 AND Mendel Error (Current) is de Novo Allele AND (Alt Allele Freq (AF) < 0.05 OR missing) AND (Effect (Combined) is (LoF, Missense)) AND (Classification is (Conflicting, Likely Pathogenic, Other, Uncertain Significance, missing)) AND Quality by Depth (QD) > 12 AND (Variant Allele Freq (Current) >= 0.35 OR missing) AND (MPC Score > 2 OR missing) AND (N of 6 Predicted Damaging is (1 of 6 Predicted as Damaging, 2 of 6 Predicted as Damaging, 3 of 6 Predicted as  Damaging, 4 of 6 Predicted as Damaging, 5 of 6 Predicted as Damaging, 6 of 6 Predicted as Damaging)) AND (PHRED > 35 OR missing) AND Essential+DD Gene? is true AND (A/Dom genes is  true OR A/Rec genes is true OR X/Rec is true OR X/Dom is true)) |
| --- |
| Dominant Filter Container |
| (Read Depths (DP) (Current) > 10 AND Genotype Qualities (GQ) (Current) > 20 AND (Mendel Error (Current) is (Transmitted, missing)) AND ((Zygosity (Father) is Heterozygous AND (Zygosity (Mother) is (Reference, missing))) OR (Zygosity (Mother) is Heterozygous AND (Zygosity (Father) is (Reference, missing)))) AND (Zygosity (Current) is (Heterozygous, Homozygous Variant, missing)) AND A/Dom genes is true AND (Alt Allele Freq (AF) < 0.05 OR missing) AND (Effect (Combined) is (LoF, Missense)) AND (Classification is (Conflicting, Other, Uncertain Significance, missing)) AND (MPC Score > 2 OR missing) AND (N of 6 Predicted Damaging is (1 of 6 Predicted as Damaging, 2 of 6 Predicted as Damaging, 3 of 6 Predicted as Damaging, 4 of 6 Predicted as Damaging, 5 of 6 Predicted as Damaging, 6 of 6 Predicted as Damaging, missing)) AND (PHRED > 35 OR missing) AND Essential+DD Gene? is true) OR (Read Depths (DP) (Current) > 10 AND Genotype Qualities (GQ) (Current) > 20 AND (Mendel Error (Current) is (Transmitted, missing)) AND ((Zygosity (Father) is Heterozygous AND (Zygosity (Mother) is (Reference, missing))) OR (Zygosity (Mother) is Heterozygous AND (Zygosity (Father) is (Reference, missing)))) AND Zygosity (Current) is Heterozygous AND A/Dom genes is true AND (Alt Allele Freq (AF) < 0.05 OR missing) AND Quality by Depth (QD) > 12 AND (Effect (Combined) is (LoF, Missense)) AND (Classification is (Likely Pathogenic, Pathogenic)) AND (Review Status is ((0 Stars) No Assertion Criteria Provided, (0 Stars) No Assertion Provided, (1 Star) Criteria Provided, Conflicting Interpretations, (1 Stars) Criteria  Provided, Single Submitter, (2 Stars) Criteria Provided, Multiple Submitters, No Conflicts, (3 Stars) Reviewed By Expert Panel)) AND Essential+DD Gene? is true) |
| Recessive Filter Container |
| (Read Depths (DP) (Current) > 10 AND Genotype Qualities (GQ) (Current) > 20 AND (Mendel Error (Current) is (Transmitted, missing)) AND (Zygosity (Current) is Homozygous Variant AND (Zygosity (Mother) is (Heterozygous, Homozygous Variant)) AND (Zygosity (Father) is (Heterozygous, Homozygous Variant))) AND (A/Rec genes is true OR X/Rec is true) AND (Alt Allele Freq (AF) < 0.05 OR missing) AND (Effect (Combined) is (LoF, Missense)) AND (Classification is (Likely Pathogenic, Pathogenic)) AND (Review Status is ((0 Stars) No Assertion Criteria Provided, (0 Stars) No Assertion Provided, (1 Star) Criteria Provided, Conflicting Interpretations, (1 Stars) Criteria Provided, Single Submitter, (2 Stars) Criteria Provided, Multiple Submitters, No Conflicts, (3 Stars) Reviewed By Expert Panel)) AND Essential+DD Gene? is true) OR (Read Depths (DP) (Current) > 10 AND Genotype Qualities (GQ) (Current) > 20 AND (Mendel Error (Current) is (Transmitted, missing)) AND (Zygosity (Current) is Homozygous Variant AND (Zygosity (Mother) is (Heterozygous, Homozygous Variant)) AND (Zygosity (Father) is (Heterozygous, Homozygous Variant))) AND (A/Rec genes is true OR X/Rec is true) AND (Alt Allele Freq (AF) < 0.05 OR missing) AND Quality by Depth (QD) > 12 AND (Effect (Combined) is (LoF, Missense)) AND (Classification is (Conflicting, Other, Uncertain Significance, missing)) AND (MPC Score > 2 OR missing) AND (N of 6 Predicted Damaging is (1 of 6 Predicted as Damaging, 2 of 6 Predicted as Damaging, 3 of 6 Predicted as  Damaging, 4 of 6 Predicted as Damaging, 5 of 6 Predicted as Damaging, 6 of 6 Predicted as  Damaging, missing)) AND (PHRED > 35 OR missing) AND Essential+DD Gene? is true) |
| Compound Heterozygous Filter Container |
| (Read Depths (DP) (Current) > 10 AND Genotype Qualities (GQ) (Current) > 20 AND (Mendel Error  (Current) is (MIE, Transmitted, de Novo Allele, missing)) AND Compound Het? (Current) is true |

| AND (Alt Allele Freq (AF) < 0.05 OR missing) AND (Classification is (Conflicting, Likely Pathogenic, Other, Pathogenic, Uncertain Significance, missing)) AND (Effect (Combined) is (LoF, Missense)) AND (N of 6 Predicted Damaging is (1 of 6 Predicted as Damaging, 2 of 6 Predicted as Damaging, 3 of 6 Predicted as Damaging, 4 of 6 Predicted as Damaging, 5 of 6 Predicted as Damaging, 6 of 6 Predicted as Damaging, missing)) AND (MPC Score > 2 OR missing) AND PHRED > 35 AND (A/Rec genes is true OR X/Rec is true OR X/Dom is true)) OR (Read Depths (DP) (Current) > 10 AND Genotype Qualities (GQ) (Current) > 20 AND (Mendel Error (Current) is (MIE, Transmitted, de Novo Allele, missing)) AND Compound Het? (Current) is true AND (Alt Allele Freq (AF) < 0.05 OR missing) AND Quality by Depth (QD) > 12 AND (Effect (Combined) is (LoF, Missense)) AND (Classification is (Likely Pathogenic, Pathogenic)) AND Review Status AND (A/Rec genes is true OR  X/Rec is true)) |
| --- |
| X-linked Filter Container |
| (Read Depths (DP) (Current) > 10 AND Genotype Qualities (GQ) (Current) > 20 AND (Mendel Error (Current) is (MIE, Transmitted, de Novo Allele, missing)) AND (Zygosity (Current) is (Heterozygous, Homozygous Variant)) AND (Segment in X: 1 - 60,000 OR in X: 2,699,521 - 154,931,043) AND (Alt Allele Freq (AF) < 0.05 OR missing) AND (Classification is (Conflicting, Other, Uncertain Significance, missing)) AND (Effect (Combined) is (LoF, Missense)) AND (N of 6 Predicted Damaging is (1 of 6 Predicted as Damaging, 2 of 6 Predicted as Damaging, 3 of 6 Predicted as Damaging, 4 of 6 Predicted as Damaging, 5 of 6 Predicted as Damaging, 6 of 6 Predicted as Damaging, missing)) AND (MPC Score > 2 OR missing) AND (PHRED > 35 OR missing) AND (X/Rec is true OR X/Dom is true)) OR (Read Depths (DP) (Current) > 10 AND Genotype Qualities (GQ) (Current) > 20 AND (Mendel Error (Current) is (MIE, Transmitted, de Novo Allele, missing)) AND (Zygosity (Current) is (Heterozygous, Homozygous Variant, missing)) AND (Segment in X: 1 - 60,000 OR in X: 2,699,521 - 154,931,043) AND (Alt Allele Freq (AF) < 0.05 OR missing) AND Quality by Depth (QD) > 12 AND (Effect (Combined) is (LoF, Missense)) AND (Classification is (Likely Pathogenic, Pathogenic)) AND (Review Status is ((0 Stars) No Assertion Criteria Provided, (0 Stars) No Assertion Provided, (1 Star) Criteria Provided, Conflicting Interpretations, (1 Stars) Criteria Provided, Single Submitter, (2 Stars)  Criteria Provided, Multiple Submitters, No Conflicts, (3 Stars) Reviewed By Expert Panel)) AND (X/Rec is true OR X/Dom is true)) |
| Failsafe Filter Container |
| ((Read Depths (DP) (Current) >= 1 OR missing) AND (Mendel Error (Current) is (MIE, Transmitted, de Novo Allele, missing)) AND (Alt Allele Freq (AF) < 0.05 OR missing) AND (Classification is (Conflicting, Likely Pathogenic, Other, Pathogenic, Uncertain Significance, missing)) AND (Effect (Combined) is (LoF, Missense)) AND (N of 6 Predicted Damaging is (1 of 6 Predicted as Damaging, 2 of 6 Predicted as Damaging, 3 of 6 Predicted as Damaging, 4 of 6 Predicted as Damaging, 5 of 6 Predicted as Damaging, 6 of 6 Predicted as Damaging, missing)) AND (MPC Score > 2 OR missing) AND PHRED > 35 AND (A/Dom genes is true OR A/Rec genes is true OR X/Rec is true OR X/Dom is true)) OR ((Read Depths (DP) (Current) >= 1 OR missing) AND (Mendel Error (Current) is (MIE, Transmitted, de Novo Allele, missing)) AND (Alt Allele Freq (AF) < 0.05 OR missing) AND (Effect  (Combined) is (LoF, Missense)) AND (Classification is (Likely Pathogenic, Pathogenic)) AND Review Status AND (A/Dom genes is true OR A/Rec genes is true OR X/Rec is true OR X/Dom is true)) |

| **Sample** | **1** | **2** | **3** | **4** | **5** | **6** | **7** | **8** | **9** | **10** | **11** | **Mean** | **Standard Deviation** |
| --- | --- | --- | --- | --- | --- | --- | --- | --- | --- | --- | --- | --- | --- |
| Clean reads | 1E+09 | 1E+09 | 1E+09 | 1E+09 | 1E+09 | 1E+09 | 1E+09 | 1E+09 | 2E+09 | 2E+09 | 2E+09 | 1.45E+09 | 74868971 |
| Clean bases (Mb) | 145914 | 127778 | 135385 | 141974 | 146927 | 146739 | 142548 | 145986 | 153738 | 152848 | 153139 | 144815.9 | 7486.8975 |
| Mapping rate (%) | 99.86 | 99.77 | 99.86 | 99.87 | 99.84 | 99.88 | 99.87 | 99.82 | 99.84 | 99.88 | 99.84 | 99.84818 | 0.0306944 |
| Unique rate (%) | 96.17 | 95.31 | 95.47 | 95.44 | 94.61 | 94.5 | 93.31 | 94.58 | 94.66 | 94.6 | 94.9 | 94.86818 | 0.7012044 |
| Duplicate rate (%) | 1.1 | 1.81 | 1.51 | 1.59 | 2.91 | 2.82 | 4.31 | 2.53 | 2.4 | 2.53 | 2.37 | 2.352727 | 0.8301707 |
| Mismatch rate (%) | 0.46 | 0.42 | 0.46 | 0.44 | 0.29 | 0.29 | 0.31 | 0.31 | 0.3 | 0.3 | 0.29 | 0.351818 | 0.0714548 |
| Average sequencing depth (X) | 49.34 | 42.8 | 45.34 | 47.62 | 48.93 | 48.92 | 46.73 | 48.55 | 51.22 | 50.92 | 51.14 | 48.31909 | 2.4738026 |
| Coverage (%) | 99.75 | 99.76 | 99.78 | 99.76 | 98.79 | 99.56 | 98.8 | 99.74 | 99.11 | 99.7 | 99.07 | 99.43818 | 0.3892141 |
| Coverage at least 4X (%) | 99.2 | 99.23 | 99.26 | 99.36 | 97.37 | 98.51 | 97.47 | 99.21 | 98.62 | 99.1 | 98.52 | 98.71364 | 0.6778881 |
| Coverage at least 10X (%) | 97.6 | 97.13 | 97.11 | 98.05 | 93.71 | 95.41 | 93.73 | 97 | 97.33 | 97.12 | 97.01 | 96.47273 | 1.4334262 |
| Coverage at least 20X (%) | 91.48 | 86.94 | 87.25 | 91.56 | 83.55 | 85.38 | 81.67 | 87.56 | 91.05 | 89.57 | 90.84 | 87.89545 | 3.2067014 |

**Supplementary Table 4a**: Sequencing metrics of embryo samples.

**Supplementary Table 4b**: Sequencing metrics of couple samples.

| **Sample** | **1** | **2** | **3** | **4** | **5** | **6** | **7** | **8** | **9** | **10** | **Mean** | **Standard Deviation** |
| --- | --- | --- | --- | --- | --- | --- | --- | --- | --- | --- | --- | --- |
| Clean reads | 1.2E+09 | 1.3E+09 | 1.4E+09 | 1.5E+09 | 1.5E+09 | 1.4E+09 | 1.4E+09 | 1.5E+09 | 1.5E+09 | 1.3E+09 | 1.4E+09 | 92667665.9 |
| Clean bases (Mb) | 122319 | 130622 | 144607 | 148658 | 148171 | 141254 | 142868 | 150047 | 147715 | 133085 | 140935 | 9266.76586 |
| Mapping rate (%) | 99.79 | 99.86 | 99.83 | 99.86 | 99.79 | 99.81 | 99.76 | 99.77 | 99.84 | 99.76 | 99.807 | 0.0388873 |
| Unique rate (%) | 92.97 | 93.56 | 92.49 | 93.29 | 92.86 | 93.07 | 92.94 | 93.45 | 92.73 | 91.89 | 92.925 | 0.48740241 |
| Duplicate rate (%) | 3.24 | 2.98 | 3.51 | 3.06 | 3.35 | 3.3 | 3.23 | 2.91 | 3.53 | 4.52 | 3.363 | 0.45568142 |
| Mismatch rate (%) | 0.63 | 0.56 | 0.39 | 0.39 | 0.4 | 0.43 | 0.4 | 0.37 | 0.37 | 0.49 | 0.443 | 0.08882567 |
| Average sequencing depth (X) | 40.08 | 43.13 | 47.09 | 48.7 | 48.41 | 46.18 | 46.7 | 49.17 | 48.23 | 42.89 | 46.058 | 3.03227381 |
| Coverage (%) | 99.84 | 99.13 | 99.85 | 99.14 | 99.84 | 99.17 | 99.85 | 99.18 | 99.86 | 99.17 | 99.503 | 0.36399176 |
| Coverage at least 4X (%) | 99.51 | 98.77 | 99.59 | 98.82 | 99.55 | 98.84 | 99.58 | 98.89 | 99.61 | 98.82 | 99.198 | 0.39194104 |
| Coverage at least 10X (%) | 98.67 | 98.03 | 98.74 | 98.03 | 98.68 | 98 | 98.73 | 98.1 | 98.8 | 97.95 | 98.373 | 0.37339434 |
| Coverage at least 20X (%) | 94.95 | 96.98 | 96.34 | 96.83 | 96.32 | 96.64 | 96.27 | 97.01 | 96.64 | 96.35 | 96.433 | 0.58969013 |
